# Preterm growth assessment: the latest findings on age correction

**DOI:** 10.1038/s41372-024-02202-z

**Published:** 2025-01-16

**Authors:** Seham Elmrayed, Susan Dai, Abhay Lodha, Manoj Kumar, Tanis R. Fenton

**Affiliations:** 1 Institute of Global Health and Human Ecology, American University in Cairo, Cairo, Egypt; 2 Alberta Health Services, Calgary, Canada; 3 Alberta Children’s Hospital Research Institute, University of Calgary, Calgary, Canada; 4 Department of Pediatrics, Cumming School of Medicine, University of Calgary, Calgary, Canada; 5 Community Health Sciences, Cumming School of Medicine, University of Calgary, Calgary, Canada; 6 Department of Pediatrics, Faculty of Medicine & Dentistry, University of Alberta, Calgary, Canada; 7 O’Brien Institute for Public Health, Cumming School of Medicine, University of Calgary, Calgary, Canada

**Keywords:** Paediatrics, Physical examination

## Abstract

**Objective:**

To evaluate the effect of age correction up to 36 months of age for growth assessments of extremely preterm (<28 weeks) and very preterm (28 to <32 weeks) infants.

**Study Design:**

This longitudinal analysis used data from the Preterm Infant Multicenter Growth Study (2001–2014).

**Results:**

1,416 children were included (Median gestational age = 27 weeks). Chronological age-based weight, height, and head circumference z-scores were consistently lower than those based on corrected age for all ages (0, 4, 8, 21 and 36 months) by up to −5.2 (95% confidence interval −5.4, −5.1) z-scores for length at term. Using chronological age, higher proportions of children were misclassified as having suboptimal growth (up to 72.9% misdiagnosed as stunted and 89.8% misdiagnosed as underweight at term).

**Conclusion:**

For extremely and very preterm children, age correction is required for all growth measures through 36 months of corrected age.

## Introduction

Accurate assessment of preterm infant growth is key to ensure children are provided with the appropriate medical and nutrition care to promote optimal growth and development. Preterm infants’ growth patterns can approximate growth of their term-born counterparts when the age of the former group is corrected for the extent of their prematurity [1–3]. Corrected age adjusts for the infant’s prematurity, calculated by subtracting the number of weeks born before 40 weeks of gestation from the chronological age of the infant supporting comparisons to growth and development references and standards at the same post menstrual ages [4].

Clinicians have been using age correction for decades; however, there remains conflicting advice on the appropriate criteria and duration for age correction [1, 2, 5]. It has been suggested that age correction is more important for children born early preterm compared to those born moderate or late preterm [1, 2, 5]. Performing age correction until children are 2 years of age seems to be most commonly practiced; however, some evidence indicates important misclassifications of preterm infants’ growth until 3 years of corrected age (CA) [1, 4, 6]. The type of correction, whether age should be fully or partially corrected, has also been discussed, but research remains inconclusive [1].

Previous findings suggested that lack of age correction can lead to the child being misclassified as underweight, wasted or stunted [6]. In a review that summarized relevant issues about age correction practice, obsolete data and outdated growth charts used in previous studies were highlighted as key challenges for providing current guidance and clinical care [2]. The issue of not correcting the age for prematurity extends beyond clinical care to include child growth problematic assessments in research studies [7, 8].

Several studies have observed age correction’s importance [1, 2, 5, 6, 9, 10] for assessing cognitive, language and motor outcomes in children born prematurely [1, 10]. The 1998 study of Wang and Sauve assessed the effect of age correction on growth assessments of children born prematurely; they found that over half of the preterm children were misclassified as having sub-optimal growth throughout the first three years of life if their age was not corrected for prematurity [6]. Accordingly, available evidence on age correction for assessing preterm growth is 26 years old, is based on data collected 33 to 48 years ago (1977 to 1992) and obsolete growth charts, including National Center for Health Statistics (NCHS)/World Health Organization reference (WHO) and a 1994 WHO growth reference [6]. This study aims to evaluate the effect of age correction on the assessment and plotting of preterm growth measures including weight, height/length and head circumference up to 36 months by using recent data and currently used growth charts.

## Methods

### Study sample

For this secondary analysis, infant and child data were obtained from the Preterm Infant Multicentre (PreM) Growth Study [11], which prospectively collected medical, growth, nutrition, and socioeconomic data (maternal and paternal education) from preterm infants in Calgary and Regina in Canada, between 2001 and 2014. All infants were cared for in level III neonatal intensive care units. We included infants who were born with gestational ages (GA) below 32 weeks with no anomalies. Neonatal data included GA in weeks (91% confirmed by ultrasound test otherwise maternal menstrual dates were used), birth weight (g), history of comorbidities, and duration of oxygen therapy. Growth data (weight (kg), length/height (cm), head circumference (cm)) were collected during their hospital stay and at 4-, 8-, 21- and 36-months corrected age at Neonatal Follow-up Clinic visits. Maternal and paternal data were collected at birth or during the first visit to the Neonatal Follow-up Clinic, and included age, and educational status.

### Measures and definitions

Children’s lengths/heights were measured by trained healthcare providers using length boards/stadiometer and their weights using infant or standing scales, which were regularly calibrated. Head circumference was measured using a flexible non-stretch head circumference measuring tape. Child low IQ was assessed by Wechsler Preschool and Primary Scale of Intelligence 3rd and 4th Edition (Index score < 70) at 3 years CA [12], necrotizing enterocolitis (NEC) was defined as Bell’s criteria 2 or higher, and bronchopulmonary dysplasia (BPD) as those requiring oxygen at 36 weeks [12].

Chronological age in days was calculated from the birth date and follow-up visit date. Prematurity was calculated by subtracting the infant’s GA in days from the full-term age (40 weeks = 280 days). Small for GA (SGA) was defined as birth weight below the 10th percentile based on the Fenton 2013 preterm growth chart [13]. The degree of prematurity was defined based on the WHO’s subcategories for extremely preterm (less than 28 weeks) and very preterm (28 to < 32 weeks) [14].

After 40 weeks postmenstrual age (term, 0 months CA), sex-specific standardized body size scores (z-scores) of weight, length and head circumference were calculated based on the chronological and the corrected age for each infant according to the WHO 2006 growth standards [15]. Weight-for-length and body-mass-index (BMI) z-scores were estimated using the Zscore06 Stata package [16].

The children’s underweight, stunting, wasting and overweight status were defined as binary variables according to the WHO 2008 guidance for children below 5 years [17]. The overweight category was defined as a z-score > 2 based on weight-for-length z-scores in infants up to 2 years of age and > 2 BMI z-scores for children older than 2 years of age. Stunting was defined as height for age <−2 z-scores and underweight as weight-for-age z-scores <−2. Wasting was defined as weight-for-length < −2 z-scores for children 0–2 years and a BMI z-score of <−2 for children older than 2 years. Suboptimal head growth was defined as a head circumference z-score of < −2.

### Statistical analysis

Statistical differences in the sample characteristics and growth measures among infants were compared using the paired t-test for continuous variables and the McNemar test for categorical variables given the statistical assumptions have been met. Statistical significance was set to p-value < 0.05. The weight, length/height, head circumference and BMI mean z-scores, and suboptimal and excess growth (stunting, wasting, underweight, overweight), assessed according to chronological and corrected ages, were compared for statistical differences using the paired t-test for the continuous variables and McNemar test for the categorical variables. These analyses were further stratified by sex and degree of prematurity to examine whether growth assessments by chronological versus corrected age differed across sex or prematurity groups (extremely and very preterm). Additionally, infants’ growth measures were plotted on WHO sex-specific growth charts based on corrected and chronological ages using the Canadian Pediatric Endocrine Group online plotting tools [18, 19]. We used STATA18 software to conduct the analyses [20].

### Ethics approval

Ethical approval was obtained through the Conjoint Health Research Ethics Board at the University of Calgary (ID: REB20-0702). The University of Calgary Conjoint and the Regina Qu’Appelle Health Region Health Research Ethics Boards granted a waiver of consent for this study. Parents/caregivers attending the follow-up clinics provided informed consent that their infants’ data may be included in research studies if used in aggregate. All methods were performed in accordance with the relevant guidelines and regulations.

## Results

A total of 1416 surviving infants were included in the PreM Growth Study. At 36 months CA, 22.1% of children were lost to follow-up [11]. The characteristics of the study cohort have been previously published [13]. In this cohort, 47.8% of children were female, the mean birthweight was 940 ± 218 grams, GA was 26.9 ± 1.8 weeks; 12.7% were born with SGA birthweights. 9.2% had NEC and 54.4% had BPD (Table 1). The predominant feeding in our units was bovine fortified mother’s own milk. Differences in the baseline characterises of those loss to follow up at 36 months CA are reported in Supplemental Table 1. Those infants who were not measured at 36 months corrected age had similar gestational ages, rates of SGA and early nutrition, significantly higher birthweights and rates of maternal smoking, lower parental higher education, lower rates of NEC and BPD.

**Table 1:** Study sample characteristics and comparison of baseline covariates in children born between 23 and 31 weeks of gestation (PreMGS, 2001-14, n = 1416).

Weeks of gestation	23	24	25	26	27	28	29	30	31
*N*	33	132	172	218	266	313	154	91	37
sex, *n* female (%)	16 (48.4)	62 (46.9)	88 (51.1)	103 (47.2)	120 (45.1)	138 (44.1)	83 (53.8)	46 (50.5)	21 (56.7)
Small for gestational age, *n* (%)	4 (12.1)	15 (11.3)	18 (10.4)	27 (12.3)	37 (13.9)	40 (12.8)	20 (12.9)	15 (16.4)	5 (13.5)
loWPPSIf, *n* (%)	5 (19.2)	16 (17.3)	11 (7.9)	11 (6.7)	14 (7.3)	18 (8.2)	4 (4.1)	2 (3.1)	1 (4.5)
Necrotizing enterocolitis, *n* (%)	3 (9.1)	11 (8.3)	17 (9.8)	20 (9.1)	19 (7.1)	42 (13.4)	6 (3.9)	9 (9.8)	4 (10.8)
Bronchopulmonary	15 (45.4)	69 (52.6)	93 (55.1)	134 (61.7)	152 (57.5)	165 (53.1)	69 (46.3)	50 (54.9)	16 (43.2)
dysplasia, *n* (%)									
Birth weight mean (sd)	570 (66.8)	666 (93.2)	762 (107.2)	866(155.4)	987(169.4)	1079(209.3)	1024(162.5)	1054(161.1)	1047(170.7)
IVH ≥ grade 3, *n* (%)	11 (33.3)	26 (19.6)	21 (12.2)	22 (10.1)	20 (7.5)	13 (4.1)	0	0	0
Any breastfeeding at discharge, *n* (%)	18 (86)	73 (91)	88 (81)	128 (88)	167 (95)	172 (92)	88 (93)	61 (87)	24 (88)
First enteral feeding, mean (sd)	7.6 (6.6)	5.8 (6.3)	4.8 (5.1)	3.9 (3.5)	3.1 (3.1)	2.6 (2.5)	2.7 (3.3)	2.1 (1.7)	2.5 (1.7)
Days of starting parenteral and enteral nutrition, mean (sd)	0.2 (0.6)	0.3 (0.5)	0.2 (0.4)	0.2 (0.4)	0.2 (0.5)	0.2 (0.5)	0.3 (0.6)	0.2 (0.6)	0.2 (0.4)
Length of stay (week of discharge), mean (sd)	39.9 (2.9)	38.9 (2.9)	37.8 (3.5)	37.6 (3.7)	36.9 (3.2)	37.3 (3.3)	36.2 (2.7)	36.9 (2.6)	36.9 (2.5)
Overweight at 3 years corrected age	0	2 (1.9)	3 (2)	6 (3.3)	5 (2.3)	6 (2.5)	2 (2)	0	1 (4.5)
Maternal age, mean (sd)	31.4 (6.2)	30.6 (6.1)	29.4 (5.8)	29.4 (5.7)	30.6 (5.5)	29.8 (6)	30.2 (5.7)	29.3 (5.6)	30.1 (4.9)
Maternal education > high school, *n* (%)	21 (72.4)	82 (67.7)	99 (64.2)	128 (64.9)	156 (67.2)	175 (64.5)	90 (68.1)	55 (65.4)	18 (60)
Paternal education > high school, *n* (%)	21 (75)	74 (62.1)	101 (70.1)	110 (60.1)	140 (62.5)	168 (65.1)	84 (65.1)	50 (63.2)	16 (57.1)
Maternal smoking, *n* (%)	5 (15.1)	28 (21.7)	40 (23.3)	35 (16.2)	48 (18.1)	67 (21.6)	25 (16.3)	21 (23.1)	10 (27)

Low IQ as assessed by Wechsler Preschool and Primary Scale of Intelligence (WPPSI) 3rd and 4th Edition (Index score < 70) [12], Small for gestational age, birth weight below the 10th percentile based on the Fenton 2013 preterm growth chart [13], necrotizing enterocolitis (Bell’s criteria 2 or higher), bronchopulmonary dysplasia (requiring oxygen at 36 weeks) [12].

### Differences in growth measures: mean z-scores and growth chart plots

The mean z-scores based on chronological age for weights, length/heights and head circumference measures were consistently lower than those based on the CA for all time points (0, 4, 8, 21 and 36-months corrected ages) (Table 2). These differences were highest for the 0-month group and decreased as children grew, however, remained statistically significant through 36 months of CA. When chronological age was used, the mean z-scores for weight, height, and head circumference were significantly lower at 0 months CA, differing on average by 4 to 5 z-scores from the corresponding CA z-scores that were distributed around zero (Table 2). Looking across the z-scores as the children aged from 0 to 36 months, the average values for weight and length improved towards the medians as the children caught up toward the the size of term born counterparts represented by the WHO charts. Head circumference averaged around the WHO median at all of the ages assessed including at 0 months CA.

**Table 2:** Z-scores of weights, heights, and head circumferences by chronological and corrected age (PreMGS).

	Term	4 months	8 months	21 months	36 months
**Weight, mean (sd)**					
Chronological age	−5.3 (0.9)	−2.4 (1.2)	−1.4 (1.3)	−1 (1.1)	0.7 (1.1)
Corrected age	−0.8 (0.04)	−1.1 (1.2)	−0.8 (1.2)	−0.5(1.2)	-0.4 (1.1)
Difference	−4.4 (−4.5, −4.4)	−1.3 (−1.4, −1.2)	−0.67 (−0.7, −0.5)	−0.44 (−0.5, −0.3)	−0.27 (−0.3,−0.1)
**Length/Height, mean (sd)**					
Chronological age	−6.6 (1.5)	−3.4 (1.3)	− (1.6)	−1.5 (1.2)	−1.1 (1.1)
Corrected age	−1.4 (1.5)	−1.3 (1.3)	−1.1 (1.5)	−0.7 (1.2)	−0.5 (1.1)
Difference	−5.2 (−5.4, −5.1)	−2.1 (−2.1, −1.9)	−1.4 (−1.5, −1.2)	−0.8 (−0.8, −0.6)	−0.5 (−0.5, −0.3)
**Head circumference, mean (sd)**					
Chronological age	−4.8 (1.5)	−0.8 (15.2)	−0.6 (1.9)	−0.2 (1.2)	−0.2 (1.2)
Corrected age	−0.0 (1.3)	0.8 (15.6)	0.1 (1.8)	0.0 (1.2)	−0.0 (1.2)
Difference	−4.8 (−5.1, −4.6)	−1.6 (−2.9, −0.3)	−0.76 (−0.9, −0.5)	−0.3 (−0.4, −0.2)	−0.2 −0.2, −0.1)
**Body mass index, mean (sd)**					
Chronological age	-	-	-	-	−0.1 (1.1)
Corrected age	-	-	-	-	−0.2 (1.1)
Difference	-	-	-	-	0.1 (−0.1, 0.2)

*sd* standard deviation.

*p*-values obtained from paired t-test of differences in means.

**Fig. 1:**
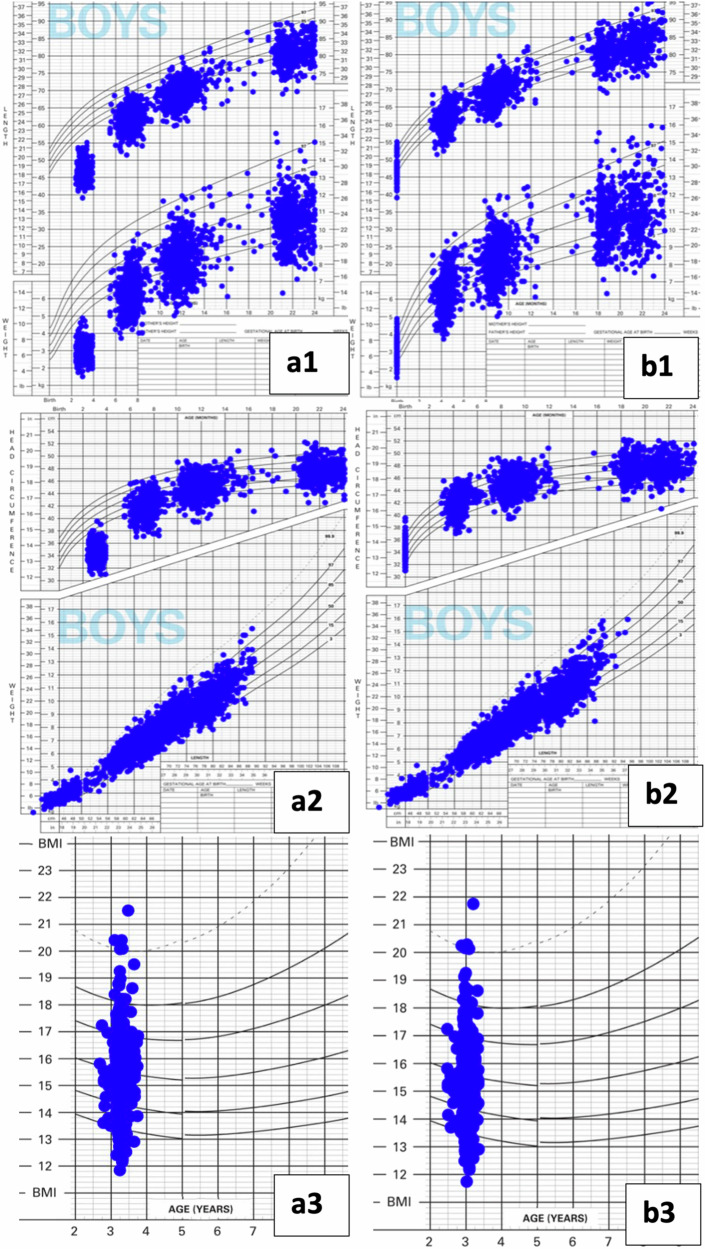
Anthropometric measurements of male preterm infants. Male preterm infants’ comparisons of plotted anthropometrics on World Health Organization charts by chronological (**a**) versus corrected ages (**b**) for the following growth measures: - a1 and b1: length and weight. - a2 and b2: head circumference and weight-for-length. - a3 and b3: body mass index.

**Fig. 2:**
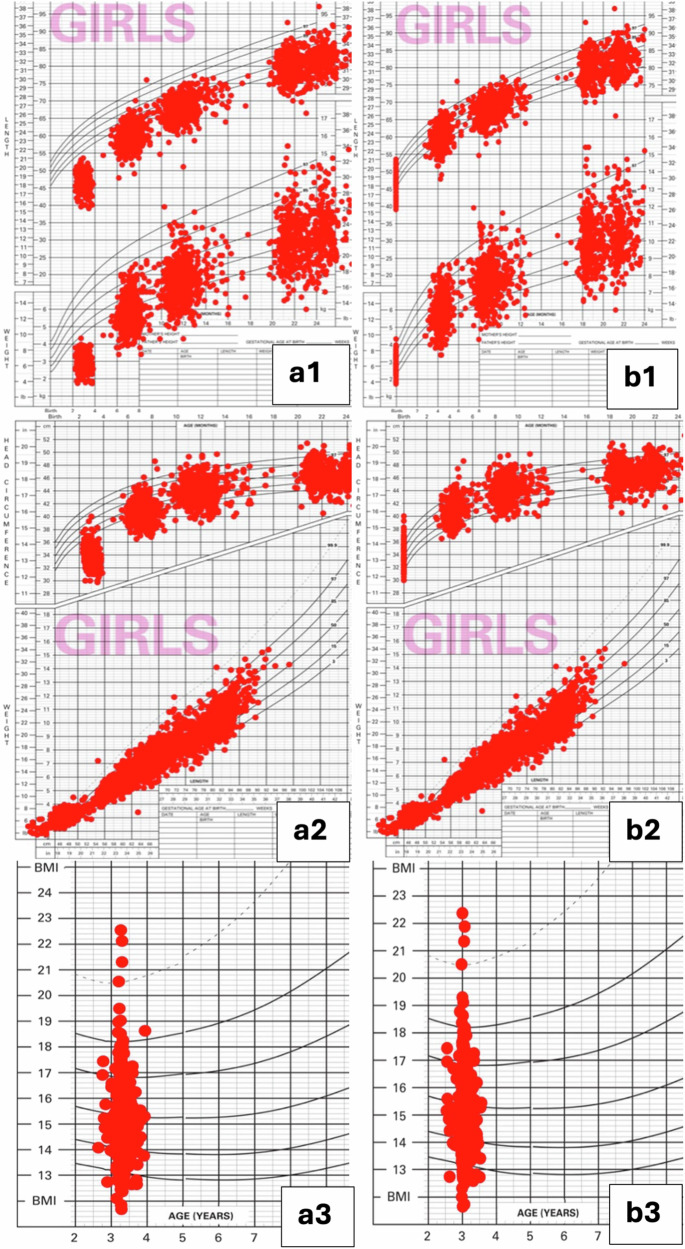
Anthropometric measurements of female preterm infants. Female preterm infants’ comparisons of plotted anthropometrics on World Health Organization charts by chronological (**a**) versus corrected ages (**b**) for the following growth measures: - a1 and b1: length and weight. - a2 and b2: head circumference and weight-for-length. - a3 and b3: body mass index.

At all ages, compared to the plotting by corrected ages, the children’s chronological age weight, length and head measurement distributions were shifted to the right (plotted at older than their true postmenstrual ages) which placed the majority of the plots below the growth curves at those older ages (Figs. 1 and 2). This shift was reflected by the mean z-scores (Table 2) and the proportions that plotted within the growth chart curves (Table 3). The differences in mean z-scores of all growth measures were consistent and did not differ across males versus females and for extremely versus early preterm groups through 36 months of corrected age (supplemental Tables 2–5).

**Table 3:** Prevalence of suboptimal and excess growth in preterm children by chronological and corrected age.

	0 months	4 months	8 months	21 months	36 months
**Stunted, height for age** < −**2 z-score,** ***n*** **(%)**					
Chronological age	401 (100%)	1103 (66.1%)	785 (64.1%)	401 (33.5%)	208 (18.9%)
Corrected age	109 (27.1%)	356 (28.3%)	288 (23.5%)	179 (14.9%)	109 (9.9%)
Percent misdiagnosed	72.9%	37.8%	40.6%	18.6%	9.0%
*P*-value	<0.0001	<0.0001	<0.0001	<0.0001	<0.0001
**Underweight, weight-for-age** < −**2 z-score,** ***n*** **(%)**					
Chronological age	585 (100%)	819 (64.7%)	422 (34.3%)	248 (20.4%)	159 (14.2%)
Corrected age	60 (10.2%)	278 (21.9%)	207 (16.8%)	145 (11.9%)	96 (8.6%)
Percent misdiagnosed	89.8%	42.8%	17.5%	8.5%	5.6%
*P*-value	< 0.0001	<0.0001	<0.0001	<0.0001	<0.0001
**Overweight, WFL** > **2 z-scores in 4**–**21 months and BMI** > **2 z-scores at 36 months,** ***n*** **(%)**					
Chronological age	79 (19.7%)	22 (1.9%)	25 (2.2%)	17 (1.5%)	21 (1.8%)
Corrected age	79 (19.7%)	22 (1.9%)	25 (2.2%)	17 (1.5%)	21 (1.8%)
Percent misdiagnosed	-	-	-	-	-
*P*-value	NA	NA	NA	NA	NA
**Wasting, WFL** < −**2 z-scores in 4**–**21 months and BMI** < −**2 z-scores at 36 months,** ***n*** **(%)**					
Chronological age	8 (2%)	40 (3.6%)	48 (4.6%)	58 (6.0%)	29 (3.2%)
Corrected age	8 (2%)	40 (3.6%)	48 (4.6%)	58 (6.0%)	36 (4.0%)
Percent misdiagnosed	-	-	-	-	0.80%
*P*-value	NA	NA	NA	NA	0.008
**Suboptimal head circumference <** −**2 z-score,** ***n*** **(%)**					
Chronological age	410 (96.9%)	294 (26.4%)	93 (8.3%)	18 (1.6%)	20 (1.8%)
Corrected age	11 (2.6%)	0	27 (2.4%)	8 (0.7%)	17 (1.5%)
Percent misdiagnosed	94.3%	26.4%	5.9%	0.9%	0.3%
*P*-value	<0.0001	NA	<0.0001	0.001	0.08

Column heading reflect time points according to corrected age.

*p*-values obtained from McNemar test of differences in proportion.

*p*-value significant at <0.05 (two sided).

*WFL* weight-for-length, *BMI* body mass index.

Wasting prevalence in children did not change when according to age correction given that the tool to assess wasting in 0–2 children (i.e., weight-for-length) does not vary by age.

### Suboptimal and excess growth

The use of chronological age put a higher proportion of the children’s length, weight and head below -2 z-scores ( < 2 percentile) at all of the ages from term to 36 months, suggesting poorer growth patterns (Figs. 1 and 2).

The estimated prevalence of suboptimal growth by chronological age versus CA varied significantly according to the age examined (Table 3). Based on the chronological age, over half the sample’s (66.1%) length growth were categorized as stunted at 4 months of age, whereas when children were assessed by their CA the proportion was less than one half (28.3%) (*p* < 0.0001, Table 3). At 36 months, 18.9% were considered stunted based on chronological age which was 9.9% after age correction (*p* < 0.0001, Table 3).

Similarly, the differences in underweight prevalence based on chronological versus CA was significant across all age groups. Underweight status in children based on chronological age was recorded in 64.7% at 4 months as compared to 21.9% after correcting children’s ages (Table 3). At 36 months, the prevalence of underweight dropped significantly from 14.2% based on chronological ages to 8.6% based on the CA.

At both 21 and 36 months, some children were misclassified when their chronological ages were used. For length/height, the use of chronological ages significantly underestimated mean z-scores by 0.4 (95% CI 0.3, 0.5) and 0.3 (95% CI 0.1, 0.3) and misclassified 19% and 9.0% at 21 and 36 months, respectively.

Significant differences were found in suboptimal head growth categorization across the ages, except at 36 months (*p* = 0.08) (Table 3). At 4 months, while none of the infants were categorized as having suboptimal head growth based on the CA, the chronological age measures classified 26.4% of children as having suboptimal head growth.

There were no differences in the assessments of wasting, overweight and obesity based on chronological and corrected ages in children 0-2 years due to the limitation of the used metric (i.e., weight-for-length), which does not vary by age. When body mass index was used at 36 months, the same proportion (1.8%) were categorized as overweight based on both chronological and corrected ages.

## Discussion

Preterm children have clinically important and statistically significant differences in all growth measures, including weight, length/height and head circumference through 36 months of CA when their growth is plotted according to their chronological age compared to their corrected ages. At 4 months of age, about 40% of extremely and very preterm infants [21] who were growing appropriately (>−2 z-scores at post term ages) [17] would be misclassified as growth faltering for weight and length if their chronological age was used for assessing their growth.

The common belief that most preterm infants are growth faltering could be at least partially attributable to the lack of proper correction for prematurity when assessing preterm growth [22–25]. Our findings show that infants born very preterm make consistent progress in catching up, with only 9.9% had height and 4% had BMI measures <−2 z scores by 36 months CA. When chronological age was used, more than twice as many children (66% vs 28%, misclassifying 38%) would have been considered stunted at 4 months of age. With regards to growth charts, the plotted growth patterns of weight-for-age, length-for-age, weight-for-length, head circumference-for-age, and age-specific BMI showed considerable differences in growth patterns according to the type of age used. When chronological age was used, the children plotted lower on the growth charts for all growth indicators, suggesting poorer growth patterns. Using corrected age provided a better representation of the plotted growth measures on the growth chart curves for weight, length and head circumference. These results confirm the need for age correction through 36 months of CA for children born extremely and very preterm to achieve accurate growth assessments.

While some very and extremely preterm infants in this study were small relative to the chart curves median at 0 months CA followed by steady catch up in length and weight to 36 months CA, their WFL categorizations suggested the opposite direction; 19.7% were categorized as overweight at 0 months CA, then at the older ages only 2.3% remained in that category. The categorization difference is likely since their lengths (27.1% stunted) at 0 months CA were lagging their weights (10.2% underweight). One could assume this data at 0 months CA suggests risk of overweight in later life. Our longitudinal analysis shows that this effect is not a prediction of later overweight but is rather a problem using a WFL metric for preterm infants prior to length catch-up, a problem also been seen from using BMI for preterm infants [26].

Preterm head circumferences catch up earlier than weight and length [27], which has been referred to as head sparing [3, 28] and has been observed to be an indicator for good development [28]. Neurodevelopmental outcomes of this cohort have been examined elsewhere [27].

The current findings are in line with those obtained by Wang and Sauve over two decades ago, who using a similar approach also observed large significant differences in growth classifications in preterm growth based on chronological versus corrected ages [6]. The need for age correction was also highlighted by literature assessing developmental outcomes in preterm populations [1, 10, 29, 30]. A recent study by Aylward indicated the need for age correction for cognitive assessments up until 2 years, and for 3 years when assessing language and motor composite scores, for all degrees of prematurity [1] to provide accurate age-appropriate assessments.

Given the complexities surrounding preterm birth, including low birthweights, immature organs and NICU exposures, children born prematurely should not be expected to mature and achieve developmental milestones faster than expected by their term-born peers [31]. While some factors can be influenced by postnatal age such as body fat deposition [32] and gastrointestinal adaptions (which is influenced by feeding) [33, 34], most aspects of childhood growth and development require correction for prematurity. Their chronological age disregards the fact that these infants are born at earlier postmenstrual ages than their term-born counterparts of the same chronological age. Age correction is grounded in the assumption that “early development proceeds as a function of time since conception” [2]. For instance, when chronological age is used to assess the growth of an infant at six months who was born four months early, the infant would be expected to have achieved the size and development of a six-month infant. When the CA is used however, the infant is considered two months of age and thus can be expected to have grown and developed accordingly. These considerable age differences can result in varying and possibly contrasting conclusions regarding the infant’s growth and developmental status, as seen in this study. We agree with D’Agostino that age correction may improve clinical capacity “to accurately recognize genuine delays as opposed to perceived delays related to a child’s gestational age at birth” [5].

In the context of childhood obesity epidemic as well as prevalent food insecurity, it is paramount that assessments of children’s growth are accurate. Misclassified infant and child growth could result in implementing additional clinical services that are not needed. When based on flawed assessments, nutrition interventions aimed at accelerating infant growth could have serious negative consequences on an infant’s health, wellbeing [35] and the feeding relationship within the family [36]. Misguided parenting practices may include over/force feeding that can disrupt children’s capacity to self-regulate their food intake [37–39], negatively influencing the child’s ability to maintain their healthy weights and have a healthy relationship with food [37–39].

Our study has a few limitations. First, this sample of extremely and very preterm infants had a variety of prenatal and neonatal morbidities and feedings that may have contributed to their growth patterns; however, our mix of patients with morbidities, a range of sizes at birth and a mix of breast and formula feeding are not unique. Accordingly, our results may be similar to results of other NICUs. Second, the anthropometric measurements were made by several healthcare providers; however, they were likely reliable since they were made by trained staff in the Follow-up Clinics [40] using regularly calibrated scales. Third, while it would be valuable to link the present findings to cardiometabolic outcomes, the required data were not available. Lastly, it was not possible to assess variations in growth classifications by age for moderate to late preterm infants as their data were not included in the PreM Growth Study dataset.

To the best of our knowledge, the study findings provide the most up-to date evidence addressing the issue of age correction in preterm growth assessments based on recent very preterm data and widely used WHO growth charts. The study sample size was sufficient to capture important statistical differences. Additionally, the plotted growth patterns on WHO growth charts provide visual evidence for the dramatic differences in growth distributions by chronological and corrected ages.

In conclusion, considering the substantial and statistically significant differences observed in preterm growth classifications according to the age used, available evidence supports the practice of age correction through 36 months of CA to avoid misclassifications of growth among extremely and very preterm children. Future research should explore age correction effects in moderate and late preterm children, and clinical guidelines should be formulated to streamline age correction in routine care to ensure optimal support for children born very prematurely.

## Supplementary information

Supplemental materials

## Data Availability

Data will be made available upon request for research purposes.
