# Supplementary material for: Preterm growth assessment: the latest findings on age correction
Source: J Perinatol. 2025 Jan 16;45(5):607–15. doi: 10.1038/s41372-024-02202-z (PMC12221983; doi:10.1038/s41372-024-02202-z)
Supplement: Supplementary file 1 — Supplemental materials [file 41372_2024_2202_MOESM1_ESM.docx]

**Supplemental Table 1**. Comparison of baseline characteristics between children with and without growth measures at 36 months corrected age (CA)

|  | Children missing all growth measures at 36 months CA | Children with at least 1 growth measure at 36 months CA | Difference  P-value |
| --- | --- | --- | --- |
| N | 309 | 1107 |  |
| sex, n female (%) | 153 (49.5%) | 524 (%) | 0.52 |
| Small for gestational age, n (%) | 36 (11.7%) | 128 (11.6%) | 0.92 |
| Low IQ, n (%) | 5 (1.6%) | 77 (7.0%) | 0.02 |
| Necrotizing enterocolitis, n (%) | 18 (5.8%) | 116 (10.5%) | 0.015 |
| Bronchopulmonary dysplasia, n (%) | 132 (42.7%) | 640 (57.8%) | <0.0001 |
| Birth weight mean (sd) | 972 (212) | 921 (219) | 0.0003 |
| Intraventricular hemorrhage ≥ grade 3, n (%) | 21 (6.8%) | 92 (8.3%) | 0.47 |
| First enteral feeding, mean (sd) | 3.4 (3.7) | 3.6 (4.0) | 0. 34 |
| Days of starting parenteral and enteral nutrition, mean (sd) | 0.2 (0.5) | 0. 3 (0.6) | 0.19 |
| Maternal age, mean (sd) | 28.8 (6.2) | 30.4 (5.7) | <0.0001 |
| Maternal education > high school, n (%) | 114 (36.9%) | 707 (63.9%) | 0.0002 |
| Paternal education > high school, n (%) | 109 (35.3%) | 653 (59.0%) | 0.002 |
| Maternal smoking, n (%) | 80 (25.9%) | 196 (17.7%) | 0.002 |

Abbreviations: sd, standard deviation

p-values obtained from paired t-test of differences in means and from McNemar test of differences in proportions.

Low IQ as assessed by Wechsler Preschool and Primary Scale of Intelligence 3rd and 4th Edition (Index score <70)(12)

**Supplemental Table 2.** Z-scores of weights, heights, and head circumferences by chronological and corrected age in females (PreMGS)

|  |  |  | |  | |  | |  |  |  |
| --- | --- | --- | --- | --- | --- | --- | --- | --- | --- | --- |
|  | **Term** | | **4 months** | | **8 months** | | **21 months** | | | **36 months** |
|  |  | |  | |  | |  | | |  |
| Weight, mean (sd) |  | |  | |  | |  | | |  |
|  |  | |  | |  | |  | | |  |
| Chronological age | -4.9 | | -2.2 | | -1.4 | | -0.9 | | | -0.7 |
| Corrected age | -0.7 | | -0.9 | | -0.8 | | -0.4 | | | -0.5 |
| Difference | -4.2 (-4.2 -4.1) | | -1.3 (-1.3 -1.3) | | -0.7 (-0.7 -0.7) | | -0.4 (-0.5 -0.4) | | | -0.3 (-0.3 -0.3) |
|  |  | |  | |  | |  | | |  |
| Height, mean (sd) |  | |  | |  | |  | | |  |
|  |  | |  | |  | |  | | |  |
| Chronological age | -5.9 | | -3.1 | | -2.31 | | -1.3 | | | -0.9 |
| Corrected age | -1.1 | | -1.1 | | -0.9 | | -0.6 | | | -0.5 |
| Difference | -4.9 (-4.9 -4.8) | | -2.0 (-2.0 -2.0) | | -1.4 (-1.4 -1. 4) | | -0. 8 (-0.8 -0.7) | | | -0.5 (-0.5 -0.5) |
|  |  | |  | |  | |  | | |  |
| Head circumference, mean (sd) |  | |  | |  | |  | | |  |
|  |  | |  | |  | |  | | |  |
| Chronological age | -4.1 | | -1.2 | | -0.6 | | -0.0 | | | -0.0 |
| Corrected age | 0.4 | | 0.3 | | 0.1 | | 0.3 | | | 0.1 |
| Difference | -4.5 (-4.6 -4.5) | | -1.5 (-1.6 -1.5) | | -0.8 (-0.8 -0.8) | | -0.3 (-0.3 -0.3) | | | -0.2 (-0.2 -0.2) |
|  |  | |  | |  | |  | | |  |
| Body mass index, mean (sd) | | |  | |  | |  | | |  |
| Chronological age |  | |  | |  | |  | | | -0.2 |
| Corrected age |  | |  | |  | |  | | | -0.3 |
| Difference |  | |  | |  | |  | | | 0.03 (0.03 0.04) |

Abbreviations: sd, standard deviation

p-values obtained from paired t-test of differences in means.

**Supplemental Table 3.** Z-scores of weights, heights, and head circumferences by chronological and corrected age in males (PreMGS)

|  | **Term** | **4 months** | **8 months** | **21 months** | **36 months** |
| --- | --- | --- | --- | --- | --- |
|  |  |  |  |  |  |
| Weight, mean (sd) |  |  |  |  |  |
|  |  |  |  |  |  |
| Chronological age | -5.4 | -2.3 | -1.3 | -0.9 | -0.6 |
| Corrected age | -0.7 | -1.0 | -0.6 | -0.4 | -0.3 |
| Difference | -4.7 (-4.7 -4.6) | -1.4 (-1.4 -1.4) | -0.7 (-0.7 -0.7) | -0.4 (-0.4 -0.4) | -0.3 (-0.3 -0.3) |
|  |  |  |  |  |  |
| Height, mean (sd) |  |  |  |  |  |
|  |  |  |  |  |  |
| Chronological age | -6.9 | -3.4 | -2.4 | -1.4 | -0.9 |
| Corrected age | -1.4 | -1.2 | -0.9 | -0.6 | -0.4 |
| Difference | -5.6 (-5.6 -5.5) | -2.2 (-2.2 -2.2) | -1.5 (-1.5 -1.5) | -0. (-0.8 -0. 8) | -0.5 (-0.5 -0.5) |
|  |  |  |  |  |  |
| Head circumference, mean (sd) |  |  |  |  |  |
|  |  |  |  |  |  |
| Chronological age | -4.9 | -1.3 | -0.4 | 0.1 | 0.1 |
| Corrected age | 0.3 | 0.4 | 0.4 | 0.4 | 0.2 |
| Difference | -5.1 (-5.2 -5.1) | -1.7 (-1.7 -1.7) | -0.8 (-0.8 -0.8) | -0.3 (-0.3 -0.3) | -0.1 (-0.1 -0.1) |
|  |  |  |  |  |  |
| Body mass index, mean (sd) | |  |  |  |  |
| Chronological age |  |  |  |  | -0.01 |
| Corrected age |  |  |  |  | -0.1 |
| Difference |  |  |  |  | 0.1 (0.1 - 0.1) |

Abbreviations: sd, standard deviation

p-values obtained from paired t-test of differences in means.

**Supplemental Table 4.** Z-scores of weights, heights, and head circumferences by chronological and corrected age in children born extremely preterm (<28 gestational weeks) (PreMGS)

|  | **Term** | **4 months** | **8 months** | **21 months** | **36 months** |
| --- | --- | --- | --- | --- | --- |
|  |  |  |  |  |  |
| **Weight, mean (sd)** |  |  |  |  |  |
|  |  |  |  |  |  |
| Chronological age | -5.3 | -2.3 | -1. 4 | -0.8 | -0.6 |
| Corrected age | -0.7 | -0.9 | -0.6 | -0. 4 | -0.3 |
| Difference | -4.6 (-4.7 - 4.6) | -1.4 (-1.5 -1.4) | -0.8 (-0.8 -0.8) | -0.5 (-0.5 -0.5) | -0.3 (-0.3 -0.3) |
|  |  |  |  |  |  |
| **Height, mean (sd)** |  |  |  |  |  |
|  |  |  |  |  |  |
| Chronological age | -6.7 | -3.4 | -2.4 | -1.4 | -0.9 |
| Corrected age | -1.3 | -1.1 | -0.8 | -0.5 | -0.4 |
| Difference | -5.5 (-5.5 -5.4) | -2.3 (-2.3 -2.3) | -1.6 (-1.6 -1.6) | -0.8 (-0.9 -0.88) | -0.5 (-0.5 -0.5) |
|  |  |  |  |  |  |
| **Head circumference, mean (sd)** |  |  |  |  |  |
|  |  |  |  |  |  |
| Chronological age | -4.8 | -1.4 | -0.6 | -0.0 | 0.0 |
| Corrected age | 0.3 | 0.4 | 0.3 | 0.3 | 0.2 |
| Difference | -5.0 (-5.1 -5.0) | -1.8 (-1.8 -1.8) | -0.9 (-0.9 -0.9) | -0.3 (-0. 4 -0.3) | -0.2 (-0.2 -0.2) |
|  |  |  |  |  |  |
| **Body mass index, mean (sd)** |  |  |  |  |  |
| Chronological age |  |  |  |  | -0.1 |
| Corrected age |  |  |  |  | -0.1 |
| Difference |  |  |  |  | 0.1 (0. 1 0.01) |

Abbreviations: sd, standard deviation

p-values obtained from paired t-test of differences in means.

**Supplemental Table 4.** Z-scores of weights, heights, and head circumferences by chronological and corrected age in children born very early preterm (28 - <32 gestational weeks) (PreMGS)

|  | **Term** | **4 months** | **8 months** | **21 months** | **36 months** |
| --- | --- | --- | --- | --- | --- |
|  |  |  |  |  |  |
| Weight, mean (sd) |  |  |  |  |  |
|  |  |  |  |  |  |
| Chronological age | -4.9 | -2.2 | -1.4 | -0.9 | -0.7 |
| Corrected age | -0.7 | -1.0 | -0.8 | -0.5 | -0.4 |
| Difference | -4.1 (-4.2 -4.1) | -1.2 (-1.2 -1.2) | -0.6 (-0.6 -0.6) | -0.4 (-0.4 -0.4) | -0.2 (-0.2 -0.2) |
|  |  |  |  |  |  |
| Height, mean (sd) |  |  |  |  |  |
|  |  |  |  |  |  |
| Chronological age | -5.9 | -3.0 | -2.3 | -1.4 | -0.9 |
| Corrected age | -1.1 | -1.2 | -1.0 | -0.7 | -0.5 |
| Difference | -4.8 (-4.8 -4.7) | -1.9 (-1.9 -1.8) | -1.3 (-1. 3 -1.3) | -0.7 (-0.7 -0. 7) | -0.4 (-0.4 -0.4) |
|  |  |  |  |  |  |
| Head circumference, mean (sd) |  |  |  |  |  |
|  |  |  |  |  |  |
| Chronological age | -4.0 | -1.1 | -0. 5 | 0. 1 | 0.0 |
| Corrected age | 0.4 | 0.3 | 0.2 | 0.3 | 0.2 |
| Difference | -4.4 (-4.5 -4.4) | -1.5 (-1.5 -1.5) | -0.7 (-0.7 -0.7) | -0. 3 (-0.3 -0. 3) | -0.1 (-0.1 -0.1) |
|  |  |  |  |  |  |
| Body mass index, mean (sd) |  |  |  |  |  |
| Chronological age |  |  |  |  | -0.2 |
| Corrected age |  |  |  |  | -0.2 |
| Difference |  |  |  |  | - |

Abbreviations: sd, standard deviation

p-values obtained from paired t-test of differences in means.

**Supplemental Table 5.** Growth classifications by chronological and corrected age in children born extremely (<28 gestational weeks) versus very preterm (28 - 31 gestational weeks) at 36 months corrected age (PreMGS

|  |  | **Extremely preterm <28** | **Very preterm 28- < 31** |
| --- | --- | --- | --- |
| **Underweight, n (%)** |  |  |  |
|  |  |  |  |
| Chronological age |  | 48 (5.3) | 44 (4.9) |
| Corrected age |  | 21 (2.3) | 27 (3.1) |
| Difference |  | <0.0001 | <0.0001 |
| **Stunting, n (%)** |  |  |  |
|  |  |  |  |
| Chronological age |  | 85 (9.6) | 56 (6.3) |
| Corrected age |  | 39 (4.4) | 30 (3.4) |
| Difference |  | <0.0001 | <0.0001 |
|  |  |  |  |
| **Wasting, n (%)** |  |  |  |
|  |  |  |  |
| Chronological age |  | 14 (1.5) | 15 (1.7) |
| Corrected age |  | 20 (2.2) | 16 (1.8) |
| Difference |  | 0.0143 | 0.3173 |
|  |  |  |  |
| **Overweight, n (%)** |  |  |  |
|  |  |  |  |
| Chronological age |  | 12 (1.3) | 9 (1.1) |
| Corrected age |  | 12 (1.3) | 9 (1.1) |
| Difference |  | - | - |

Differences based on a McNemar test of proportions.
